# Disintegrating ipseity: a gerontological exegesis of Alzheimer’s disease and relational identity in Alice Munro’s fiction

**DOI:** 10.1186/s13010-026-00217-2

**Published:** 2026-06-08

**Authors:** Farman Ullah, Shihong Du, Wen Jin, Yasir Ali Nagar

**Affiliations:** 1 College of International Studies, Southwest University, Chongqing, 400715 China; 2 School of International Chinese Studies, East China Normal University, Shanghai, China; 3 School of English Language and Literature, Nangarhar University, Jalalabad, Afghanistan

**Keywords:** Alzheimer’s, Narratology, Gerontology, Identity, Fiction

## Abstract

**Background:**

Alzheimer’s is a progressive disorder of the brain that gradually affects memory, personality, behaviour, identity, and interpersonal relationships, particularly among older adults. Alice Munro’s novella “The Bear Came Over the Mountain” offers a compelling literary framework for exploring these impacts through narrative gerontology, a lens that emphasizes storytelling’s role in shaping identity and relational bonds during ageing and illness.

**Method:**

This study applies narrative gerontology to analyze Munro’s depiction of Alzheimer’s, focusing on the experiences of Fiona and Grant. Through detailed textual analysis, it examines the gerontological concept of “we-narrative” (shared relational identity) to understand how identity, memory loss, and relational dynamics evolve.

**Results:**

Munro portrays Alzheimer’s as both a medical condition and a social experience, fragmenting Fiona’s sense of self and reshaping the couple’s shared identity. Fiona’s fading memories unravel her narrative, diminishing her autonomy, while Grant’s shift from husband to caregiver redefines their relationship. The novella’s disjointed structure reflects the cognitive chaos of Alzheimer’s, deepening its emotional impact. The gerontological “we-narrative,” representing shared relational identity, reveals the enduring strength of their bond, as Grant’s selfless actions highlight a transformed expression of love.

**Discussion:**

Dementia narrative features how caregivers’ identity gradually forms through the lived experience of supporting a loved one with cognitive decline. These findings align with narrative gerontology’s view that stories help individuals and caregivers navigate identity and loss. Munro’s work challenges stereotypes about ageing, emphasizing the emotional complexity of caregiving and the resilience of human connections.

**Conclusion:**

Munro’s novella powerfully illustrates Alzheimer’s multifaceted effects, using narrative to explore identity, care, and relational adaptation in the context of ageing and illness.

## Introduction

Alice Munro’s fiction, “The Bear Came Over the Mountain,” published in the 2001 collection *Hateship*,* Friendship*,* Courtship*,* Loveship*,* Marriage*, presents an elderly couple encountered with wife’s progressive Alzheimer’s and its repercussions. According to Elizabeth Baikie, “there has been relatively little research on the impact of dementia on marriage” [1]. Therefore, the impact of Alzheimer’s on one of the spouses in a dementia narrative needs to be explored. Taking on the role of caregiver for a partner living with Alzheimer’s, particularly within ageing narratives, often brings profound emotional, relational, and psychological strain. In the same vein, Cristina Garrigós examines “Alzheimer’s is often associated with old age” [2]. Narratively speaking, depictions of Alzheimer’s provide a powerful lens for exploring distinctive challenges. These include the abrupt loss of professional identity and earning capacity during working years, highlighted strains on family relationships due to unexpected caregiving demands and acute financial pressures stemming from premature income cessation. Moreover, individuals with Alzheimer’s frequently encounter disbelief or dismissal from family members in “Alzheimer’s narrative” [3], who may question or reject the diagnosis because of the widespread misconception that Alzheimer’s affects the elderly. Anyone with Alzheimer’s faces cumbersome health-related issues and arduous challenges in life.

These health-related issues, whether physical or mental, need to be vigilantly attended by the government, communities or individuals [4–7] to prevent further deterioration and “reduce the risk of dementia” [8]. Alzheimer’s locates the issue of suffering, loss and forgetting at the centre of lived experience. In *Dementia and Literature*, Tess Maginess explores Medical/Health humanities and the necessity for studies of cultural representations of diseases such as Alzheimer’s [9]. As it shapes identities, relationships and understanding of ageing beyond the medical frame. A progressive deterioration in cognitive function due to damage, ageing, or disorder in the brain has gravely affected older adults. Dementia, with Alzheimer’s disease as one of its common forms, is advancing at a rapid pace in various countries around the world, especially in the United Kingdom. Whether written or spoken literature about “Alzheimer’s” brings forth a sense of anticipatory terror, horror, and fear that is popular in contemporary English fiction, Media, TV drama series and films. Alzheimer’s disease is a state which “primarily affects the elderly and, by 2015, the number affected by this disease will have almost trebled in less than twenty years” [10]. Surprisingly, the progressive erosion of brain cells, Alzheimer’s Disease also concerns what might particularly affect, including language, loss of memory, judgement and problem-solving.

Furthermore, Alzheimer’s Disease is gravely depicted in literary works, reflecting the growing recognition of humanity’s value in gerontology over the past two decades. This interdisciplinary approach emphasises how cultural narrative can complement medical understanding of dementia in relation to ageing [11]. Influential critics such as Diane Gruber and Kathleen Woodward have established a prominent role for the humanities in ageing studies [12, 13]. This very assertion is backed by Sally Chivers, who argues that “Humanities scholars who have turned to age studies… have exposed theories of – “modes of conceptualizing” – old age, opening the door for new models. They acknowledge and encourage the social construction of age and work toward understanding the processes behind it” [14]. In the same vein, Chivers extends that “literary gerontology can balance social and cultural narratives of ageing with the physical dimensions of ageing to develop rich models for new understandings of late life” [14]. Building on this foundation, our analysis examines the intimate connection among narrative dementia, ageing, illness, decay and disability [15]. Alzheimer’s asachronic condition tied to ageing across cultures [16]. Our study focuses on how Alzheimer’s-induced memory erasure dismantles personal identity and a sense of belonging within intimate relationships, particularly the gerontological “we-narrative” (shared relational identity) of marriage. In Munro’s “The Bear Came Over the Mountain,” Fiona’s fading memories fracture her selfhood and dissolve the couple’s joint history with Grant, while paradoxically fostering new affective ties—such as her bond with Aubrey—and silencing once-central elements of her past. Understanding such literary portrayals of “dementia in literature, film, mass media and social media in order to better understand cultural stigma related to dementia” [17]. Hence, engaging with the narrative of people living with Alzheimer’s often experience “emotional strain, loss of self... silences in the archive, [and] the loss of verbal communication in dementia” [18].

Rebecca Anna Bitenc (2012) explores that the literary representations of dementia enhance our understanding of what it is like to experience this disease. According to Cristina Garrigós, “Alzheimer’s is often associated with old age” [2]. In literary work elderly with “dementia are forced to witness the decline of their own mental faculties, the loss of language and of memory... all that is intimately bound up with their personal identity and that supposedly defines what it is to be human” [19]. In the same vein, individuals with early-onset Alzheimer’s frequently face disbelief or dismissal from family members, who may question the diagnosis that why Alzheimer’s affect elderly often.

Old age is “the frailty of our bodies, the most disabling feature of aging is the frailty of our minds” [cited in 1]. Hence, Alzheimer’s disease gravely appears in old age as “Alzheimer’s disease does proportionally affect more people over 60 than below 60” [20], which is examined in Alice Munro’s novella “The Bear Came Over the Mountain”, is a poignant example of narrative fictional potential to capture the relational and psychological impact of Alzheimer’s. Through the experiences of Fiona, who suffers from Alzheimer’s, and her husband, Grant, in “The Bear Came Over the Mountain”, explore how ageing and illness disrupt memory, identity, autonomy, and relational bonds. The story goes beyond clinical symptoms, framing Alzheimer’s as a socio-medical construction of old age. According to Anne Davis Basting, “we fear dementia... because a diagnosis of dementia triggers thought” [21]. Old age’s social construction of Alzheimer’s is all trouble and sorrow [22, 23]. As Fiona’s Alzheimer’s progresses, her memories fade, erasing pieces of her identity and altering her once-stable relationship with Grant. Munro’s portrayal invites readers to view Alzheimer’s as a shared experience of loss and transformation for both the individual and their loved ones. As Basting (2009) mentioned, “people... see dementia as more than a death sentence” [21].

This lack of research into the way that the sufferer, as well as the person who looks after the victimised with Alzheimer’s disease, is “vulnerable to the debilitating effects of negative self-stereotyping and stereotype threat” [24]. Hence, “Understanding Alzheimer’s Disease, though a disease with very real and often terrible effects on the material body, is also experienced in relation to its cultural construction and its intimate connection with the representation of ageing as decline, makes clear the urgency of interrogating the narratives that make up this discourse, and this includes literary texts” [20]. In the light of this urgency, this paper offers a close reading of Alice Munro’s “The Bear Came Over the Mountain” to illuminate the relational and psychological dimensions of Alzheimer’s. The analysis begins with the novella’s narrative technique, third-person limited narration with internal focalization primarily through Grant, before applying the framework of narrative gerontology. This approach ensures that interpretations remain anchored in Munro’s literary poetics as Grant’s partial, self-justifying perspective restricts direct access to Fiona’s inner experience, mirroring the opacity of dementia, while fragmented syntax, temporal shifts, free indirect discourse, and ironic understatement evoke cognitive disarray and underscore ethical ambivalence in caregiving and relational transformation.

Narrative gerontology then provides the conceptual lens for understanding these textual dynamics, particularly how shared relational identity (we-narrative) persists or fractures amid memory loss. As Tollefsen and Gallagher (2016) examine, “We-narratives can be told from the first-person plural perspective (we) or the third-person plural perspective (they), and they can be told by individual narrators or by a process of joint narration and co-construction. Like individual narratives, they can be told from the inside or the outside—by individuals within a group or by an individual outside a group” [25]. In this gerontological sense, “we-narrative” refers to the shared stories that stabilize joint agency and couple identity—not a literal narrative voice in the text. Close reading of Munro’s focalization and the literary analysis take priority, revealing conceptual and ethical tensions rather than merely illustrating gerontological points. These gerontological concepts offer a novel interpretive lens for Munro’s novella, complementing the existing body of criticism that has long examined its themes of ageing, dementia, and relational dynamics.

### Critical studies of “The Bear Came Over the Mountain”: ageing, dementia and relational dynamics in Munro

“The Bear Came Over the Mountain” has been discussed widely. As Muhammad Chowdhury Islam (2011) explores in “The Bear Came Over the Mountain,” there exists a “bizarre relationship between two unacquainted families… [whose members] suffer from two different types of trauma: psychic hysteria and physical immobility” [26]. Additionally, the story marks people, events, landscape, and era that shape a woman’s identity as she journeys from adolescence to adulthood. According to Sara Jamieson (2014), Alice Munro’s long short story explores “the spatial and social complexities” of the elderly [27]. Pavlína Studená’s (2024) article examines how Alice Munro’s deliberate use of indeterminacy and narrative gaps in “The Bear Came Over the Mountain” actively engages readers in hypothesis-building and interpretive revision, while briefly noting similar ambiguity in Sarah Polley’s (2006) film adaptation “Away from Her” [28]. According to Casado-Gual Nuria, the story examines the elderly spouse’s “social and psychological facets of memory in connection with late-life expressions of love... the complexities of old age, dementia and gender difference” [29].

In *Reading Alice Munro*,* 1973–2013*, Robert Thacker mentioned that Alice Munro provides an overview of the topics she was researching and writing about:

I see this story as paralleling pretty well the experiences of the Irish immigrants of that time—the traumatic beginning, bewildering and difficult struggles, the nearly paralyzing disappointments (James’ death), then the slow prosaic adjustment and absorption into the country’s life. There can’t be any spectacular ‘making it’ in the new land because the Irish usually didn’t get that far. They remained mostly working class, lower middle-class, or self-sufficient farmers. [30]

Robert Thacker examines that Alice Munro “gives us a glimpse of her methods. She has been reading emigrant letters” which in turn is reflected in Munro’s writing as Munro writes about “desperate, ill-prepared, sickly passengers, the familiarity with death, terrifying inroads of the fever, the despair of those taken to the quarantine sheds on Grosse Isle” [30].

Additionally, Robert Thacker, in his comprehensive biography, *Alice Munro: Writing Her Lives* (2005; updated 2011), situates the novella within Munro’s personal and artistic trajectory, highlighting its prescient exploration of cognitive decline—echoing her mother’s Parkinson’s disease and foreshadowing her own later dementia—while underscoring the story’s nuanced portrayal of marital loyalty amid infidelity and loss, themes that resonate with the relational transformations examined here. Alice Munro’s life is a ‘journey through the intricate tapestry of human experience, woven with delicate precision and profound insight’; however, themes which are explored in Munro’s writing include memory loss, gender, identity, mortality, human fragility, human complexity, and rural life.^1^ Magdalene Redekop’s feminist analysis in *Mothers and Other Clowns: The Stories of Alice Munro* (1992), though predating the novella, offers valuable insights into performative female agency and ironic self-presentation in Munro’s fiction, which can be extended to Fiona’s ambiguous dementia behaviours as a form of resilient, if fragmented, identity negotiation rather than mere decline. Besides, Magdalene Redekop focuses on Alice Munro’s obsession with mothering and relates it to the hallucinatory quality of her magic realism [31].

Furthermore, Brenda Pfaus’ critiques of Munro’s character psychology and moral ambiguities (e.g., in her analyses of psychological realism in Munro’s earlier works) illuminate Grant’s flawed evolution from unfaithful husband to sacrificial caregiver, emphasizing the novella’s refusal to sentimentalize or condemn ethical compromises in the face of illness. Brenda Pfaus gives a very lively and fresh insights into some of Alice Munro’s work, which includes, *Lives of Girls and Women* (1971), *Who Do You Think You Are?* (1978), *Dance of the Happy Shades* (1968), *Something I’ve Been Meaning to Tell You* (1974), and *The Moons of Jupitor* (1982).^2^ Additionally, Brenda Pfaus’ structural focus on the transition to adulthood, the depiction of problems and the tension between social expectations and personal experience [32].

Moreover, Oriana Palusci, in her edited volume *Alice Munro and the Anatomy of the Short Story* (2017) and related essays, explores “intralinguistic, interlinguistic and intersemiotic translation in “The Bear Came Over the Mountain” (2001) and in its film adaptation *Away from Her/Lontano da lei* by Sarah Polley (2008), pinpointing shifts and losses implied by each distinctive translation process, from English into Italian, from page to screen” [33]. Oriana Palusci’s focus on translational shifts in representing fluid and complex narratives of dementia, institutional care, and emotional resilience^3^—perspectives that align with this paper’s emphasis on narrative as a tool for reconstructing identity and bonds. Thus, “The Bear Came Over the Mountain” uses narrative elements to reflect the psychological, social and relational dimensions of Alzheimer’s, which affect not only Fiona’s mental state but also her correlative relationship with Grant. By dialoguing with Kathleen Woodward, Sally Chivers, Sarah Falcus, Brenda Pfaus, Magdalene Redekop, Cristina Garrigós, and Robert Thacker, our analysis extends their literary focus through narrative gerontology, addressing an underexplored dimension: how storytelling not only reflects but actively enables adaptation and meaning-making in the shared experience of Alzheimer’s, thus enriching Munro criticism with interdisciplinary insights into ageing as a relational and narrative process. By applying narrative gerontology as a theoretical framework, this study explores how Munro’s short long story addresses ageing as both a biological and a social process. Moreover, prior critical engagements with Alice Munro’s “The Bear Came Over the Mountain” have richly illuminated its thematic depths, particularly in relation to ageing, memory fragility, and relational ethics, providing a foundation upon which this study’s narrative gerontological lens builds.

### Literary representations of Alzheimer’s

There are various ways to explore the representations of Alzheimer’s in cultural narratives. The representation of Alzheimer’s in literature enables us to examine the disease in terms of

the profound ontological issues it raises about our very being as individual “selves”, about how this self operates relationally– how does the individual engage with and separate from “society” and, at the most profound level, what it means or ought to mean, to be human. [cited in 2]

A person with Alzheimer’s features issues such as focus on the carer’s problem, loss of memory related to loss of self, personhood, etc [2]. A significant amount of study has conducted on Alzheimer’s in the last decade, while there is also a very great interest in the representation of Alzheimer’s in fiction in relation to ageing, as it is evident from articles published internationally^4^ and also books such as Sally Chivers’s *The Silvering Screen: Old Age and Disability in Cinema* (2011), Cristina Garrigós’ *Alzheimer’s Disease in Contemporary US Fiction* (2022), Pamela H. Gravagne’s *The Becoming of Age: Cinematic Visions of Mind*,* Body and Identity in Later Life* (2013), Martina Zimmermann’s *The Poetics and Politics of Alzheimer’s Disease Life-Writing* (2017) and Raquel Medina’s *Cinematic Representations of Alzheimer’s Disease* (2018).

Critics such as Robert Thacker, Lucy Burke, Brenda Pfaus, Tess Maginess, Sarah Falcus, Katsura Sako, and Rebecca Bitenc have published widely on literary representations of Alzheimer’s. These studies are essential for comprehending the intimate relationship between literature and the disease. As mentioned by Lucy Burke (2015), “Alzheimer’s is not simply the name of a disease, it is the term around which a whole assemblage of problems and possibilities circulate. Crucially,… [Alzheimer’s is] a form of cultural production in its own right” [37]. Bruke refers to Alzheimer’s memoirs.... Alzheimer’s narratives and Alzheimer’s film. In the same vein, Burke states that we are in the “Age of Alzheimer’s” [37]. Similarly, Garrigós (2022) also fill the gap by highlighting that “insufficient attention has been paid to dementia patient narratives” [2].

In *Contemporary Narratives of Dementia: Ethics*,* Ageing*,* Politics* (2019), Sarah Falcus and Katsura Sako examine different types of Alzheimer’s in contemporary narrative fiction. The book is structured by genre, encompassing auto/biographies, fictional illness narratives, detective fiction, family and inheritance novels, and children’s picture books. This structure reflects the authors’ keen awareness of both the ethical imperative to represent dementia and the potential risks inherent in such portrayals; mindful of the negative stereotypes and stigma surrounding the condition, they advocate for “awareness of dementia” that highlights the broader social influence and transformative potential of literary texts [38].

Rebecca Bitenc’s *Reconsidering Dementia Narratives: Empathy*,* Identity*,* and Care* (2019) explore essential role of narrative in fostering innovative approaches to understanding, engaging with, and caring for individuals living with Alzheimer’s. It also examines how stories about dementia in narrative fiction, including B.S. Johnson’s *House Mother Normal*, J. Bernlef’s *Out of Mind*, Margaret Forster’s *Have the Men Had Enough?*, Michael Ignatieff’s *Scar Tissue*, Kazuo Ishiguro’s *The Unconsoled*, and Lisa Genova’s *Still Alice*. Additionally, the book includes life writing, film, and other media—both mirror and actively influence societal perceptions of the condition. Rebecca Bitenc’s emphasizes the importance of embodied and relational dimensions of identity in dementia, while proposing ways in which narratives shape, inform and enhance care practices. At the same time, it critically challenges the assumption that narrative-induced empathy inevitably leads to more compassionate or human care. Through a cross-medial, interdisciplinary lens, the analysis encompasses auto/biography, graphic narrative, novels, film, documentary, and collaborative storytelling, using case studies to delineate the ethical limits and possibilities of narrative studies in a medical humanities framework to ethical representation and care [39].

Additionally, understanding Alzheimer’s disease and its intimate relationship with ageing in literature is a debated topic over the last two decades [40, 41]. The existing literature has extensively examined the narrative technique to depict psychological aspects of dementia and memory loss, particularly highlighting the individual’s cognitive decline. According to Robert Wilson, “the pathological diagnoses of Alzheimer’s disease and other common conditions that impair cognition” [42]. Moreover, works by critics like Kathleen Woodward (1999) and Sally Chivers (2011) have emphasized the value of humanities and gerontology [13, 43], the emphasis has often been on cultural representations and social stereotypes surrounding old age rather than on how narrative itself functions as a means of coping, connection, and identity reconstruction for those impacted by “Alzheimer’s disease (AD)” that cause memory loss [44].

Alzheimer’s disease causes much more than just memory loss as one of its most serious but often overlooked effects is the deep isolation many older people with the condition face due to a lack of ongoing community support and public recognition of their needs [45]. Despite growing awareness of these social challenges, relatively little research explores how narrative can act as a therapeutic and interpretive resource—not only for individuals with Alzheimer’s but also for their caregivers. This leaves a significant gap in understanding how storytelling, as seen in Alice Munro’s “The Bear Came Over the Mountain,” can reveal the combined realities of memory decline, shifting identity, and changing relationships through the framework of narrative gerontology. Furthermore, Chivers’ (2011) work draws attention to the ways in which the elderly are stereotyped or marginalized: while literature and film often stereotype or marginalize the elderly, there is comparatively less interest in the potential literature has to refract ageing and illness as more complex social processes than the natural decline implied by biology. In this paper, we address this gap by introducing narrative gerontology. This approach not only help readers to comprehend psychiatric disease in Munro's fiction but also demonstrates how memory loss rebuilds life and personal identity from the inside out.

### Narrative gerontological concept of “we-narrative”: disruption, loss and relational transformation

Narrative gerontology’s concept of “we-narrative” illustrates how Grant sustains a relational shared history. Tollefsen and Gallangher (2016) note that such narratives foster “stability and depth” in “shared agency” [25]. This relational “we” emerges through third-person focalization on Grant, whose memories reconstruct their bond amid Fiona’s decline. In the same vein, according to Tollefsen and Gallangher (2016), “we-narrative” can be “the third-person” that constructs shared relational identity [25]. In “The Bear Came Over the Mountain”, Alice Munro employs this approach by focusing on the shared history of Grant and Fiona, constructing a “we-narrative” that underscores their collective identity even as Fiona’s memory loss fragments her sense of self. Hence, the story utilizes a sophisticated ‘we’ that act as a bridge between the elderly couple’s intimate relationship and the readers, such as the description of the nursing home (Meadowlake) is not just a place for Fiona but it also represented as a space that highlight the universal, inescapable decline of the body and mind that await everyone as “the narrator’s point of view that readers perceive the reality of the literary space created by the short story” [46]. For instance, as they drive to Meadowlake, Fiona recalls a skiing trip: “[O]h, remember. Only it was in the moonlight” [47]. This shared memory offers a moment of continuity, as it connects them to a time when their relationship was full of vitality, even while they faced the uncertainty of Fiona’s condition.

Narrative gerontology underscores “we-narratives” in intimate relationships and represents shared identities [25]. As Peter Goldie (2012) defines, a narrative is “something that can be told or narrated... a representation of those events which is shaped, organized, and coloured, presenting those events, and the people involved in them, from a certain perspective or perspectives, and thereby giving narrative structure—coherence, meaningfulness, and evaluative and emotional import— to what is related” [48]. This narrative loss becomes evident as Fiona’s affection shifts toward Aubrey, dissolving the foundation of her bond with Grant. Munro narrates that he had thought of asking her to come home, but he knew he couldn’t do that, which underscores the dissolution of their shared identity and Grant’s new, solitary role. Scholl and Sabat (2008) highlight how such disruptions challenge relational identities, portraying Alzheimer’s as a shared crisis [24].

The gerontological concept of “we-narrative” can “construct a collective subject” [49], referring to the shared relational identity of the couple that emerges through joint biographical storytelling, rather than a first-person plural narrative voice in the text. In “The Bear Came Over the Mountain”, Grant’s perspective often blends nostalgia with present realities, reflecting his struggle to maintain this collective “we” despite Fiona’s decline. He recalls their nightly routine before her illness as “[t]his was their time of liveliest intimacy, though there was also, of course, the five or ten minutes of physical sweetness just after they got into bed” (p. 5). Such recollections emphasize the shared rituals that defined their partnership, even as Fiona’s growing attachment to Aubrey challenges its cohesion. Grant’s internal reflections suggest that their shared identity is no longer mutual, as Fiona’s memory loss shifts her focus away from their marriage to her new reality.

This disruption of the “we-narrative” reflects not only memory loss but also the erosion of shared identity within the couple [50]. Munro’s fiction portrays the profound relational transformation as Fiona’s progressive memory erosion shifts their long marriage from mutual reciprocity and shared affection to an asymmetrical dynamic centred on care and adaptation. For example, her confusion about basic shared history—“[S]he asked Grant when they’d moved to this house. ‘Was it last year or the year before?’ ‘It was twelve years ago,’ he said. ‘That’s shocking (p. 7)”—marks the gradual erosion of their joint foundation, leaving Grant to hold onto the past alone.

As Fiona develops a new emotional connection with Aubrey at Meadowlake, previously intimate gestures are redirected, for instance, when “[s]he tapped her fingers across the back of his hand (p. 9),” a small act that once belonged to Grant. This redirection stirs jealousy and grief in Grant, yet he comes to accept that Fiona’s current comfort and sense of belonging in her present environment take precedence over preserving an unchanging past. The change challenges conventional marital expectations but simultaneously demonstrates the flexibility of the relational bond when confronted with illness.

Grant’s ultimate response highlights this transformative resilience through sacrifice. Instead of resisting Fiona’s attachment, he takes a difficult step of contacting Aubrey’s wife, Marian, to ask her to allow Aubrey back to Meadowlake periodically. He reasons, “[C]ould she consider taking Aubrey back to Meadowlake, maybe just one day a week, for a visit? It was only a drive of a few miles” (p. 15). This selfless act redefines love from mutual procession and desire to compassionate service and prioritization of others’ happiness, even at personal cost. Munro thus shows how Alzheimer’s dismantles certain relational elements while enabling new, adaptive forms of commitment and empathy.

These adaptive expressions of love and commitment—manifested through Grant’s sacrificial intervention and Fiona’s redirected intimacy—exemplify how narrative gerontology functions in practice to navigate relational disruption of dementia. By actively preserving, reinterpreting and enacting their shared history through reflection, intentional care, and acceptance of change, Grant sustains a transformed “we-narrative” that upholds meaning and connection despite Fiona’s cognitive fragmentation. This aligns with contemporary scholarship emphasizing narrative approaches as a mechanism for reconstructing relational identity, “memory, and personal identity, [that is] highlighting the role of narrative” [51] as well as relational bonds in dementia caregiving. In Munro’s novella, this approach is vividly enacted as Grant’s deliberate acts of care and acceptance not only preserve fragments of their shared past but actively reshape their relational identity into a form of enduring compassion. Such portrayals affirm narrative gerontology’s value in transforming the crisis of dementia into opportunities for meaningful continuity and renewed connection, beyond mere cognitive loss. These narratives pose relational, ethical and personal belonging questions about identity crisis, fragmentation, care and cognitive decline that help us to interrogate the cultural discourse of dementia.

In this light, the gerontological concept of “we-narrative” illustrates how Grant’s steadfast commitment to Fiona sustains their relational bond despite her memory loss. When Fiona expresses confusion about her life, Grant reframes his role within their shared narrative, ensuring that their collective history remains alive, at least for him. This dynamic is poignantly expressed in Fiona’s final words to Grant when she says “[Y]ou could have just driven away without a care in the world and forsook me. Forsooken me. Forsaken” (p. 24). Here, Fiona’s fragmented speech reflects her cognitive decline, but the “we” perspective persists as Grant anchors her fading identity through his presence and his devotion to their shared story. Munro’s use of narrative gerontology highlights the resilience of human connections, even as they are tested by the challenges of ageing and memory loss.

In a nutshell, Munro’s story demonstrates how Alzheimer’s leads to a reconfiguration of relational bonds and a transformation of love. Despite Fiona’s diminishing memory, Grant’s commitment to her persists, as he continues to visit and support her, even though she rarely recognizes him. This enduring loyalty underscores the resilience of relational bonds, even when the foundation of memory has been stripped away. As Chivers (2003) notes, narrative gerontology sheds light on the relational dimension of identity, showing how relationships can transform and persist in new forms. Furthermore, Grant’s journey reflects an evolution from husband to caregiver who values Fiona’s comfort above his own emotional needs, capturing the transformative impact Alzheimer’s has on relationships. Munro’s portrayal invites readers to see Alzheimer’s not only as a disease but as a catalyst for relational change, redefining love, loyalty, and identity within the space of shared suffering.

### Memory as core to selfhood

There is an intimate relationship between “self and memory” [52]. In the same vein, Ann Davis Basting, in her seminal book, *Forget Memory* (2009), questions how people with dementia are impacted by the fear of memory loss, as it is equal to the loss of self. People with dementia are “nearly drowning in fear, grief, anxiety, and anger” as well as loss of self [21]. Hence, the relationship between self and memory is intimately tied up with our past, as what is narrated [53, 54]. Accordingly, “memory has long been linked to the construction of the identity” [2]. Memory serves as the foundation of Fiona’s identity in “The Bear Came Over the Mountain,” intertwining her self-perception with her relationship to Grant when she asks “[D]o you think it would be fun if we got married?” (p. 1). This query reveals not mere forgetfulness but a profound fracturing of her personal narrative and shared history. Similarly, Grant observes Fiona’s resilience in notes when he recalls that “[o]ver a year ago, Grant had started noticing so many little yellow notes stuck up all over the house. That was not entirely new. Fiona had always written things down—the title of a book she’d heard mentioned on the radio or the jobs she wanted to make sure she got done that day” (p. 2). Fiona uses to maintain control amid her slipping memory. Even her morning schedule is meticulously documented: “ [7] A.M. yoga. 7:30–7:45 teeth face hair. 7:45–8:15 walk. 8:15 Grant and breakfast” (p. 24). As Anne Davis Basting (2003) explains, such depictions prompt deeper inquiries into selfhood:

Understanding how Alzheimer’s is perceived and represented can help interrupt and change the experience of the disease for those who suffer, those who anticipate suffering, and those who care for its victims. Understanding the depiction of the self in the crisis of Alzheimer’s can also teach us the meaning and value of the ‘‘whole’’ self. Exactly how does one achieve a ‘‘self?’’ Who are we without memory? Is a ‘‘self’’ possible when the ability to construct narrative through memory is broken? [55]

These are not mere questions but rather responses to those victimised by Alzheimer’s disease. Furthermore, scholars have observed the relationship between memory and identity, particularly in Alzheimer’s patients. Kathleen Woodward argues that memory is not merely a cognitive function but “a narrative construct that holds together a person’s sense of self” [13]. As Fiona loses her memories, she becomes a fragmented version of herself, unable to hold onto the narrative threads that once defined her identity. She also “[w]ent to town and phoned Grant from a booth to ask him how to drive home (p. 2),” which is a pivotal moment when Fiona’s memory loss intrudes into her daily routines. Narrative gerontology, as Sally Chivers (2003) asserts, offers insights into how storytelling shapes identity. Through Fiona’s deteriorating memory, Munro poignantly demonstrates the role of narrative in maintaining identity; without memory, Fiona’s personal narrative unravels, leaving her disoriented and estranged from herself and “[S]he would stand in doorways trying to figure out where she was going. She forgot to turn on the burner under the vegetables or put water in the coffeemaker” (p.25).

Serious attention should be paid to the social, psychological, behviroul and relational identity of an elderly person with progressive “Alzheimer’s disease and other dementias” [56]. As Fiona’s condition progresses, and her autonomy diminishes, signifying a profound shift in her self-concept [57]. Munro captures this vulnerability when Fiona, no longer shopping alone, as “[A] policeman picked her up as she was walking down the middle of the road, blocks away” (p. 3). Her confusion peaks in moments like asking “[W]hy am I here? (p. 3)”, underscoring the erosion of her agency. In the same vein, Martina Zimmermann (2010) reinforces that “Alzheimer’s disease is the most common neurodegenerative disorder in today’s developed world that is also increasingly picked out as a focal theme in fictional literature.” As it deals “with the subjectivity of human experience” [58]. Hence, Alzheimer’s features loss of memory, loss of self and “behavioural changes” [59]. This loss extends beyond the individual, reshaping relational dynamics, particularly in Fiona and Grant’s marriage. As Fiona’s illness advances, Grant transitions from husband to caregiver, redefining their shared identity through acts of support amid her growing dependence.

### Identity fragmentation as well as Grant’s identity shift from husband to caregiver in narrative techniques

Over the past two decades, Dementia and identity crisis have emerged as a central concept in literature, sociology and psychology, where they are understood as a dynamic, socially constructed which influence behaviour, self-perception, and role performance [60]. This conceptual field encompasses role identity theory, which posits that individuals derive self-meaning from the roles they occupy (e.g., spouse, caregiver), and identity change occurs through transitions, stressors, or external demands that disrupt prior role enactments. In the context of chronic illness, caregiving research builds directly on these foundations by examining how the burdens of long-term care—physical, emotional, and social—erode caregivers’ well-being while prompting profound identity reconstruction [61]. Spousal caregivers, in particular, experience role strain as the marital partnership shifts toward asymmetrical dependency, often leading to diminished self-efficacy, burnout, and a redefinition of the self from equal partner to primary protector [62]. Munro’s narrative style complements the theme of identity disintegration through its fragmented structure and the use of disjointed dialogue, evoking Fiona’s cognitive disarray. For example, her faltering speech and nonsensical moments reflect internal confusion, aligning with Sarah Falcus’s (2014) observation that “literary depictions of Alzheimer’s disease often use fragmented narratives to mirror the cognitive and psychological experience of memory loss” [20]. In this way, Munro’s techniques visually and emotionally represent the fragmentation that patients endure, as narrative gerontology posits that storytelling is integral to identity amid such decline. This fragmentation also impacts caregivers, whose identities are reshaped by stress and burden [61]. In the novella, Grant’s transformation from husband to caregiver illustrates this, as the ageing population increases demands on relatives, profoundly altering daily lives. In the same vein, Garrigós mentioned, “Alzheimer’s affects our idea of the identity of… person[s] and their sense of belonging to a group” [2].

Much attention has been given to identity in the past two decades. Identity is one of the most popular interests because of its influential impact on individual behaviour and attitude [60]. Caregivers’ physical and mental health, as well as their identities, are gravely affected due to the cumbersome stress and arduous burden [61], as the patients are hard to look after and cured without any pressure [63]. In “The Bear Came Over the Mountain”, Grant undergoes a profound transformation as he transitions from being Fiona’s husband to becoming her caregiver, while “spouse can adversely affect the caregiver’s emotional and physical health” [61]. This shift is deeply tied to the ageing population and the prevalence of people with chronic illness or disability who need some type of intermittent or long-term care. As Eifert et al. (2015) note, “most of the care responsibility will fall on relatives, such as spouses and adult children, and profoundly change their day-to-day lives” [62]. This observation directly reflects Grant’s experience as he confronts Fiona’s memory loss and ultimately makes the difficult decision to move her to Meadowlake, a long-term care facility. Caregiver identity changes to a great extent and leads to long-term exhaustion and diminished interest as well [64]. Early in their relationship, Grant’s identity as a husband was intertwined with his intellectual pursuits and, later, his infidelities. As Yilmaz and Gundogar note, “providing care for an impaired older adult can place the caregiver at risk for negative physical, emotional and social outcomes” [64]. Despite his transgressions, he always defined himself as Fiona’s partner, stating: “[H]e had never stopped making love to Fiona. He had not stayed away from her for a single night” (p. 6). This assertion reveals his belief in the permanence of their bond, even as his actions sometimes undermined it. However, Fiona’s declining health forces Grant to confront his guilt and reassess his role in their marriage, moving from partner to protector.

Furthermore, Fiona’s memory deteriorates, and Grant tries hard to “deal with difficult emotions such as grief, fear, resentment, anger, guilt, and helplessness” [62] to help her without any physical or mental change. However, Grant’s caregiving role becomes both practical and emotional, marking a significant change in his identity. He initially struggles to navigate the institutional rules of Meadowlake, such as not visiting Fiona for the first thirty days: “[A]round the third or fourth day they would start lamenting and begging to be taken home… Grant phoned every day and hoped to get the nurse whose name was Kristy” (pp. 4–5). This shift from equal partner to concerned visitor underscores Grant’s sense of helplessness and his attempt to maintain some semblance of their relationship. His daily calls to Kristy highlight his growing preoccupation with Fiona’s well-being, signalling his transition to a more nurturing role.

In addition, Grant’s identity shift is solidified as he prioritizes Fiona’s happiness over his own desires. When Fiona forms a deep emotional attachment to Aubrey, a fellow resident, Grant initially feels excluded and jealous. However, he ultimately decides to back Fiona up to reconnect with Aubrey, once after Aubrey is removed from Meadowlake as “[G]rant realized he’d failed with Aubrey’s wife… Being up against a person like that made him feel hopeless, exasperated, finally almost desolate” (p. 21). Grant’s desperation to alleviate Fiona’s suffering leads him to consider Marian, Aubrey’s wife, as a possible romantic partner, solely to ensure Aubrey’s return to Meadowlake. This self-sacrificial act reflects a profound evolution in Grant’s identity, as he redefines love as an act of service and compassion, rather than possession or desire. By the story’s end, Grant fully embodies the role of caregiver, demonstrating an emotional growth rooted in his unwavering commitment to Fiona’s happiness.

However, Grant’s shift from husband to caregiver represents a reconfiguration of his own identity, as he navigates the complexities of supporting Fiona without the mutual recognition of their shared history. Munro poignantly portrays this transition when Grant contemplates asking Fiona to come home but ultimately decides against it, recognizing her contentment with Aubrey. The spouse and her caregiver are simultaneously interacting identities—the identity of the caregiver is deeply rooted in the spousal relationship. Additionally, Henderson (2001) proposes that care can take place within a relationship [65], and that is a pivotal moment in Grant’s transformation when he realizes that his primary role is now to support Fiona’s well-being rather than maintain his spousal connection. Moreover, this change reflects T. McLaughlin’s assertion that caregivers often experience an identity crisis while they navigate their relationships with Alzheimer’s patients [66]. For Grant, this crisis is particularly painful, as it requires him to relinquish his role as Fiona’s partner and redefine his identity through caregiving and emotional sacrifice. Furthermore, Munro’s story captures the strain Alzheimer’s disease places on intimate relationships, particularly marriage. Grant’s identity is tied to his role as Fiona’s husband, but as her memory fades, he struggles to maintain this role in the face of her affection for another man, Aubrey. Munro illustrates Grant’s internal conflict as he observes Fiona’s growing attachment to Aubrey; he could have done something then, but instead, he got up and left. This line encapsulates Grant’s sense of helplessness and highlights the emotional distance Alzheimer’s creates within the marriage. According to Elizabeth Baikie (2002), one of the spouses’ caregivers perceived “strain and depression [... while] caring for partners with dementia” [1].

Simon Chester Evans contends that Alzheimer’s forces caregivers into complex emotional roles, often challenging their own identities and expectations [67]. Munro’s depiction of Grant’s journey aligns with this view, illustrating how his role shifts from partner to observer and ultimately to a caregiver who makes difficult sacrifices for Fiona’s happiness. By accommodating Fiona’s emotional needs and supporting her relationship with Aubrey, Grant redefines his place within the marriage, accepting a relational dynamic dictated by her illness.

## Conclusion

An elderly “individual[s] living with dementia is deprived of core functions of his or her personality” and memory [68]. In “The Bear Came Over the Mountain”, Munro employs third-person limited narration focalized primarily through Grant to portray Alzheimer’s inexorably dismantles memory, selfhood, and relational bonds while simultaneously opening a narrative lens onto the stories that arise from psychological and emotional aspects of progressive cognitive decline as Fiona said “[I]’m just losing my mind” (p. 3). Fiona’s gradual estrangement from her past self and from Grant, presented only through Grant’s partial recollections, mirrors a narrative of deepening isolation and adaptation, yet the story resists pure fatalism by foregrounding resilience, empathy, and transformed expressions of care. Through these textual strategies—fragmented syntax, temporal dislocation, free indirect discourse, and implicit irony—Munro masterfully Munro highlights Alzheimer’s not merely as a biological decline but as a shared human crisis that reshapes lives with dramatic intensity and emotional depth. Using narrative gerontology as a framework, we foreground how memory loss alters both individual and shared identities by perturbing the ways people narrate themselves and tell the stories about the group.

In the novella, Alzheimer’s caregiver identity emerges as the caregiver role intensifies, through “playing detective” to manage uncertainty and ‘reshaping identities’ in a shifting context [69]. Emotional sacrifice and redefined roles, Grant’s journey into the future of memory and identity tells an increasingly human tale of the resilience of humans and human connection. The story treats Alzheimer’s as a shared crisis that shifts the emphasis from the decline of individuals and relationships to how ageing and illness occur as common experiences requiring empathy, adaptation and even storytelling.

### Future research direction

Future research should extend the application of narrative gerontology to interrogate the sociocultural dimensions of Alzheimer’s disease across diverse literary corpora, particularly those representing marginalized or non-Western perspectives. Investigating how narratives construct and contest ageing and cognitive decline in postcolonial or indigenous contexts could illuminate alternative paradigms of care and identity. Additionally, comparative analyses of literary and cinematic depictions of Alzheimer’s might elucidate how medium-specific techniques shape perceptions of relational dynamics and caregiver experiences. Exploring the therapeutic potential of narrative interventions, such as storytelling workshops for patients and caregivers, could further bridge humanities and clinical practices, fostering resilience and meaning-making. Such inquiries would enrich interdisciplinary understandings of Alzheimer’s as a shared human experience, challenging hegemonic gerontophobic discourses and advocating for inclusive, culturally nuanced frameworks of support.

## Data Availability

No datasets were generated or analysed during the current study.

## Footnotes

1 https://fullpdfword.org/reviews/u32GB9/244534/4968440-alice-munro-writing-her-lives.

2 For more detail see Brenda Pfaus’ 1984 book, *Alice Munro*, in which Pfaus critically examines Alice Munro’s early career works, as Brenda explores how female identity, small-town domestic realities, and autobiographical narrative are narrated.

3 *Away From Her* (Italian: *Lontano da lei)*, is a 2006 Canadian adapted drama film. It was theatrically released in US 2007, and Italy 2008. It based on “The Bear Came over the Mountain,” a poignant drama about married couple facing the challenges of Alzheimer’s disease.

4 See the work by Edward Drott [34], Atsushi Asai and Fukuyama [35], and Wearing [36] in order to know how Alzheimer’s is shaped in cultural narratives.
